# Pattern of Pediatric Supracondylar Fracture Operated at A Rural Teaching Hospital of Nepal: A Descriptive Cross-sectional Study

**DOI:** 10.31729/jnma.4869

**Published:** 2020-03-31

**Authors:** Poojan Kumar Rokaya, Dhan Bahadur Karki, Mangal Rawal, Deoman Limbu, Suryaman Menyangbo, Harihar Devkota

**Affiliations:** 1 Department of Orthopedics & Trauma Surgery, Karnali Academy of Health Sciences, Jumla, Karnali, Nepal; 2 Department of General Surgery, Karnali Academy of Health Sciences, Jumla, Karnali, Nepal

**Keywords:** *children*, *fracture*, *humerus*, *supracondylar*

## Abstract

**Introduction:**

Supracondylar fracture of humerus is one of the common pediatric fractures encountered in our daily clinical practice. The purpose of this study is to determine the pattern of supracondylar fracture operated at rural teaching hospital of Jumla, Karnali Nepal.

**Methods:**

A descriptive cross sectional study was conducted at Jumla, Karnali after Institutional Review Committee approval. Operating room notes from 15 May 2017 to 16 November 2019 were retrieved to gather the following information: patients address, age, sex, side, injury mechanism, displacement, neurovascular injury, concurrent injuries, initial management by traditional bone setters, time between injury and surgery, operative technique. Data analysis was done using Statistical Package for Social Sciences version 20.

**Results:**

Left side predominated with 88 (63.7%) and extension type was common in 135 (97.8%). Thirteen (9.4%) patients were initially managed by traditional bonesetters. A total of 138 children underwent operative fixation with mean age of 7.47 years and gender ratio of 2:1 boy to girl. Fall from cliff, ladders and rooftops were the prevailing cause of injury 73 (52.8%). Average time between injury and surgery was 5.2 days. Closed reduction was done in 100 (72.4%) patients whereas open reduction was necessary in 38 (27.5%) patients.

**Conclusions:**

Closed extension type pediatric supracondylar fracture was common in this study. Fall from cliff, rooftop and ladder are the major cause of fracture. Delayed presentation and initial management of the fracture by the traditional bonesetters makes supracondylar fracture more challenging in resource limited setting like ours.

## INTRODUCTION

Supracondylar fracture of humerus is one of the commonest fractures encountered in our daily clinical practice. They account for 50% to 70% of all pediatric elbow fractures and represent approximately 17% of all paediatric fractures.^1^ It occurs primarily between 5-8 years of age with equal incidence among males and females.^2^ About 98% of these fractures are classified as extension type and usually result from fall onto outstretched hand with the elbow in full extension and wrist in dorsiflexion.^3^ Most fractures occur on the left side with posteromedial displacement being common in 75 % of the cases.^4^

Immediate neurovascular injury and potential complications like cubitus varus deformity, compartment syndrome, Volkmann ischemic contracture and trochlear osteonecrosis make supracondylar fracture a serious injury.^5^ Delayed presentation due to lack of transport, uneven geographical topography, massage by a traditional bonesetter and negligence by the parents has made supracondylar fracture more challenging in our setup. The current trend in our hospital for the treatment of displaced supracondylar fractures is closed reduction percutaneous pinning (CRPP), when closed reduction fails with three attempts open reduction internal fixation (ORIF) is performed.

The purpose of this study is to determine the pattern and treatment characteristics of paediatric supracondylar fracture operated at rural teaching hospital of Jumla, Karnali Nepal.

## METHODS

After Institutional Review Committee approval (IRC No.076/077/03) single center descriptive cross sectional study was conducted at Karnali Academy of Health Sciences teaching hospital Jumla, Nepal. Operating room notes from Jestha 2074 to Kartik 2076 (15 May 2017 to 16 November 2019) over a period of 30 months were retrieved to gather the following information: patients address, mode of injury, age, sex, side, extension or flexion mechanism, displacement, closed or open fracture, neurovascular injury, concurrent injuries, initial management by traditional bone setters, time between injury and surgery, CRPP or ORIF, approach for ORIF and intraoperative complication. Children age ≤14 years managed surgically with CRPP or ORIF for supracondylar fracture with complete records was included in the study. Exclusion criteria included: Patient age >15 years, incomplete medical record and supracondylar fracture managed conservatively in plaster slab. Dataanalysis was done using Statistical Package for Social Sciences (SPSS Inc. version 20, Chicago, Illinois) to find out the mean, average and range.

## RESULTS

**Figure 1:**
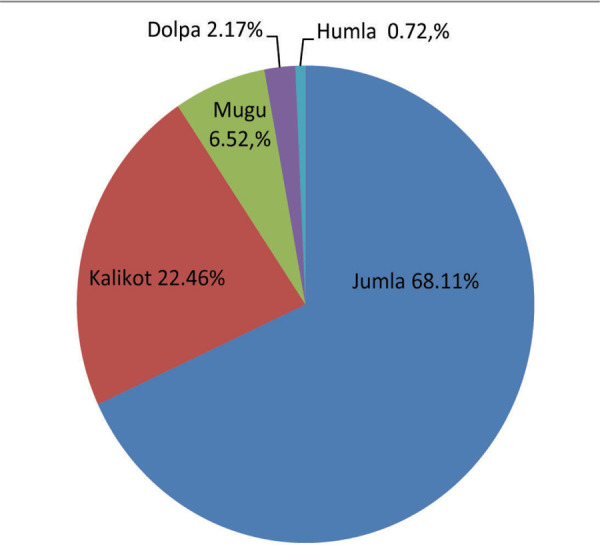
Geographic distribution of patients.

Altogether 138 children were managed surgically for supracondylar fracture over a period of 30 months. The geographical distribution of patients is shown (Figure 1).

The demographic data including patient and fracture pattern is mentioned (Table 1).

**Table 1:** Patient and fracture pattern.

Variables		n(%)
Sex	Male	93 (67.3)
	Female	45 (32.6)
Side	Right	50 (36.2)
	Left	88 (63.7)
Mode of injury	Fall while playing	57 (41.3)
	Fall from cliff	34 (24.6)
	Fall from ladder	22 (15.9)
	Fall from rooftop	17 (12.3)
	Fall from horse	3 (2.1)
	Fall from bicycle	3 (2.1)
	Fall from ox	2 (1.4)
	Fracture type Open	4 (2.8)
	Close	134 (97.1)
	Mechanism Flexion	3 (2.1)
	Extension	135 (97.8)
Gartland type	Type II	17 (12.5)
	Type III	118 (87.4)
Displacement	Posterolateral	39 (28.8)
	Posteromedial	96 (71.1)
Nerve vascular injury	Brachial artery injury	1 (0.7)
	Median nerve injury	5 (3.6)
	Ulnar nerve injury	2 (1.4)
Concurrent fractures	Ipsilateral radius and ulna fracture	4 (2.8)
	Ipsilateral clavicle fracture	1 (0.7)
	Ipsilateral radius and proximal humerus fracture	1 (0.7)

In this study boy to girl ratio was 2:1 with a mean age of 7.47 years (Range 2-14 years). Fall from cliff, ladders and rooftops were the major mode of injury accounting for 52.8%. Fracture occurred predominantly on the left side with 63.7%. Approximately 135 (97.8%) patients had extensor mechanism with posteromedial displacement being common in 96 (71.1%) patients. There were 134 (97.1%) closed fractures and four (2.8%) open fractures. Neurovascular injury was seen in seven (5%) patients. Concurrent fracture was found in six (4.3%) patients. Thirteen out of 138 (9.4%) patients were initially managed by traditional bonesetters and faith healers. Average duration from day of injury to day of surgery was 5.2 days (Range 2-14 days). The pattern of operative technique is tabulated (Table 2). CRPP was done in 100 (72.4%) patients whereas 38 (27.5%) patients required ORIF.

**Table 2:** Operative technique.

Variables		n(%)
Operative technique	ORIF	38 (27.5)
	CRPP	100 (72.4)
Approach for ORIF	Anterior	1 (2.6)
	Medial	9 (23.6)
	Posterior	28 (73.6)

## DISCUSSION

This study reflects the epidemiological parameters of pediatric supracondylar fracture operated at a rural orthopedic setup of Nepal. All the patients belonged to Karnali province. Majority were permanent resident of Jumla (68.11%) followed by Kalikot (22.46%), Mugu (6.52%), Dolpa (2.17%) and Humla district (0.72%). In this study, the mean age of the patient was 7.47 years (2-14 years) which is similar to other studies.^6,7^ Male outnumbered female with 2:1 male to female ratio. Our gender ratio is in consistent with the study conducted by Okubo et al although recent studies have found nearly equal incidence among both sexes.^8^ Fracture occurred predominantly in the left side involving 63.7% of total cases in this study. Protective posture assumed by the non-dominant extremity during fall could be the likely cause. Fall onto outstretched hand from cliff, rooftop and ladder were the common mode of injury observed in this study accounting for 52.8% of total falls. Children occasionally climb cliff to cut grass for household purpose in mountain districts like Jumla, Kalikot, and Mugu. Rooftops in the remote villages are usually open and lack protective bars or railings. Wooden ladders present in houses are devoid of side bars in most of the rural community of Karnali province. Extension type of fracture was noted in 135 (97.8%) patients whereas flexion type was noted in three (2.1%) patients which is comparable with previous studies.^9,10^ Posteromedial displacement of the distal fragment was seen in approximately 71.1% of patients in our series which is compatible with other studies.^11^ Four (2.8%) patients presented with open fracture in this series which coincides with the study conducted by Mangwani et al.6 Preoperative neurovascular injury was documented in seven (5%) patients. Median nerve was injured in five patients whereas two patients had ulnar nerve injury. One patient with open fracture had median nerve injury and pink pulseless hand. Pulse returned upon fixation of fracture via anterior approach. L.V. Barr in his series of 159 supracondylar fracture reported neurovascular injury in six (3.7%) patients.^12^ Concurrent fractures were found in six (4.3%) patients in this study. Four patients had ipsilateral fracture of the distal third of forearm bones, one patient had ipsilateral clavicle fracture and one patient had ipsilateral fracture of distal radius and proximal humerus. Concurrent fractures in pediatric supracondylar fracture have been reported up to 5% in the literature.^13^ Average duration between day of injury and day of surgery was 5.2 days (2-14 days). Delayed presentation is not uncommon in our hospital due to lack of transport, uneven geographical topography and initial management by traditional bonesetter. In this study, 13 (9.4%) patients were initially managed by traditional bonesetters and faith healers. Lack of awareness, poverty and unavailability of nearby health facility could have promoted faith healers and traditional bone setters in remote areas of lower income country like ours.^14,15^ Moreover, negligence by the parents to seek medical advice has made supracondylar fracture more challenging in our rural community which could alter the outcome of management.

CRPP was successful in 100 (72.4%) patients whereas ORIF was inevitable in 38 (27.5%) patients. The rate of ORIF for supracondylar fracture in published literature ranges from 1.3% to 46%.^16^ Closed reductions usually fails when there is massive elbow swelling, low lying fracture, pillar communition, positive pucker sign and Gartland Type IV supracondylar fracture.^17,18^ Delayed presentation could be one of the potential causes for failure of closed reduction in our study. Among 38 patients who underwent ORIF, 28 patients were approached through posterior triceps splitting approach, 9 patients through medial approach and one patient with neurovascular injury through anterior approach. Surgical approach for ORIF was decided as per operating surgeon's preference, experience and fracture personality. There is paucity in the literature regarding the standard approach which brings about better functional and radiological outcome with minimal complications. Open reduction via posterior approach is easy to perform, provides access to both the cortex at a time but it is associated with higher rate of loss of ROM and trochlear osteonecrosis.^19^ The merits of medial approach are lesser chance of iatrogenic ulnar nerve injury and unsightly scar.^20^ The preferred surgical approach should allow for anatomic reduction, access to involved neurovascular structures, satisfactory cosmetic and functional outcomes with minimal complications.^21^

Our study was dependent solely upon operating room data which is one of the major limitations. We could not include postoperative complications and treatment outcome because of poor patient compliance in regular follow up.

In our opinion this is the first rural population based study in Nepal to investigate the pattern of pediatric supracondylar fracture. Multi centric study should be conducted in other rural parts of the country to validate this study.

## CONCLUSIONS

Closed extension type of pediatric supracondylar fracture was common in this study. It was prevalent in boys and predominantly involved the non-dominant side. Fall from cliff, rooftop and ladder are the major preventable cause of fracture in the rural community of Karnali Nepal. Delayed presentation makes supracondylar fracture more challenging in resource limited setting like ours. Initial management of the fracture by the traditional bonesetters could alter the outcome of surgical treatment.

We recommend the stakeholders to take initiation in preventing falls by securing the rooftops and ladders. Traditional bone setters and faith healers should be counseled to minimize the morbidity. Government should raise awareness in the rural community regarding preventable injuries, establish health facility in remote parts and strengthen the existing health referral system.

## Conflict of Interest:

**None.**
